# Photonic/Electronic Material Performance and Application Based on Nanocrystals and Nanostructures

**DOI:** 10.3390/nano13172460

**Published:** 2023-08-30

**Authors:** Eun-Cheol Lee

**Affiliations:** 1 Department of Nano Science and Technology, Graduate School, Gachon University, Gyeonggi 13120, Republic of Korea; eclee@gachon.ac.kr; 2 Department of Physics, Gachon University, Gyeonggi 13120, Republic of Korea

Electronic, optoelectronic, and optical devices have become integral to the fabric of the modern life, underpinning critical advancements in information technology, energy utilization, biotechnology, environmental monitoring, and nanotechnology. In the relentless pursuit of superior device performance, an imperative task emerges—the exploration of fresh frontiers in optical and electronic material properties, with a focus on their applicability within these devices. Within this context, nanocrystals and nanostructures emerge as compelling candidates, seamlessly integrated into devices while endowing them with unique physical properties, distinct from their bulk counterparts.

We present to you this Special Issue of Nanomaterials dedicated to exploring the remarkable advancements in the realm of photonic and electronic materials based on nanocrystals and nanostructures. This compilation of cutting-edge research and review articles explores innovative methods for enhancing nano-scale materials in the realms of energy storage, power generation, mechanical properties, stability, and optical sensing.

The pursuit of efficient energy storage solutions has never been more critical, and in our first article, Velmurugan et al. delve into the realm of Ni and Sn perovskite NiSn(OH)_6_ nanoparticles [1]. Through meticulous exploration, they demonstrate the synergistic effects of combining Ni^2+^ and Sn^4+^ ions, resulting in hexahydroxide nanoparticles with enhanced energy storage capabilities. The insights gained from this study offer a promising avenue for the development of next-generation energy storage applications.

Triboelectric nanogenerators (TENGs) offer a unique approach to harvesting mechanical energy, and the work of Kim et al. takes this concept further by incorporating an intermediary layer of gallium-based liquid metal particles [2]. Their investigation into the use of Galinstan particles within TENGs not only contributes to the fundamental understanding of energy conversion mechanisms, but also opens doors for potential applications in wearable devices, sensors, and biomedical fields.

Mechanical properties play a pivotal role in performance of materials, and Lee et al. explore the influence of mucin on the mechanical properties of vesicles [3]. Their atomic-force-microscope-based investigation uncovers intriguing insights into the interplay between mucin and dipalmitoylphosphatidylcholine (DPPC), presenting a potential platform for exploring plasma membrane anchoring and cellular signaling.

Perovskite nanocrystals have captivated researchers for their optoelectronic properties, and Liu and Lee provide a comprehensive review of advancements in perovskite nanocrystal stability enhancement [4]. The authors discuss the exciting potential of CsPbBr_3_ perovskites while addressing challenges posed by environmental factors, offering valuable guidance for researchers seeking to harness the full potential of perovskite nanomaterials.

Lastly, Rajamanikandan et al. present a detailed review on the optical sensing of toxic cyanide anions using noble metal nanomaterials [5]. This work underscores the significance of nanoscience and nanotechnologies in enabling sensitive and accurate detection of harmful elements in environmental water samples, with various optical sensing approaches promising a better understanding of water toxicity.

The collective contributions in this Special Issue represent a significant stride in advancing the field of nanomaterials and their applications. We extend our heartfelt gratitude to the authors for their exceptional research and to the peer reviewers for their valuable insights that have enriched the quality of these articles. We hope that this compilation sparks new ideas, encourages collaborations, and inspires further investigations into the vast potential of nanocrystals and nanostructures in the realm of photonic and electronic materials. As we journey through these pages, let us be reminded of the transformative power of nanotechnology in shaping the future of science and engineering.
